# Headache of Several Years’ Standing Cured by Natrum-muriaticum

**Published:** 1895-12

**Authors:** Joseph T. O’Connor

**Affiliations:** New York


### HEADACHE OF SEVERAL YEARS' STANDING CURED BY NATRUM-MURIATICUM.

JOSEPH T. O'CONNOR, M. D., NEW YORK.

On September 22d, 1893, Mrs. M. came to me at the instance of Dr. Joseph Schmitz, of this city. She complained of headache that had begun several years ago and has been recurring with increasing frequency, so that now it comes at least once a week, and as it keeps her away from business for two and sometimes three days at a time it is making serious inroads upon her financial status.

The pain is in the occiput and left temple, but when it is very bad there is pain all over the body with inability to move, even the fingers, at which time there is a crushing pain in the joints.

She sweats very easily and any exposure to the air then brings on an attack of headache. During the headache, light aggravates very much, but by covering up the head and causing it to perspire she gets a little relief. Any jar or noise or talking aggravates. Appetite is good; bowels in good condition; circulation good; has cramps in the legs at night frequently. Belladonna was given. In a week she returned with the statement that she was better at first but that the headache had occurred every other day. On this hint I inquired about her earlier history, and found that she had been brought up in a malarial district and that for years she, with the rest of her family, had been dependent upon Quinine to keep down the "chills." Prescribed Natrum-mur.$^{30}$, two doses, with placebo. She never had another headache and the cure has remained permanent. Her greatest astonishment is that she should have been cured of such a long lasting trouble that all allopathic measures failed to influence, by two sets of little powders.

